# A European “SDGs community” to act together: The Italian initiative

**DOI:** 10.7189/jogh.12.03016

**Published:** 2022-05-14

**Authors:** Raffaella Bucciardini, Anna M Giammarioli

**Affiliations:** National Centre for Global Health, Istituto Superiore di Sanità, Rome, Italy

## WHERE WE ARE

Health protection and health promotion still represent a major challenge for policymakers across the world. The outbreak of the COVID-19 pandemic has further highlighted the structural weaknesses already existing within and between countries, which, depending on the country, may concern the fragility of health systems, the vulnerability of economic systems, or the fragility of the government, which have determined the difficulty of coping with the virus and the ability to mitigate the emerging health crisis.

The 2020 Human Development Report showed that the impact of the pandemic on a global scale is higher than any other major disease. The Human Development Index, which combines life expectancy, education, and national income, is facing an unprecedented decline since its introduction in 1990 [1].

However, this new health emergency also offers the opportunity to take serious actions to put health and health equity at the core of the National Recovery and Resilience Plan (NRRP). What we learned is how deeply the world is interconnected, how health is globalized, and how we are all facing the same challenges, although in different contexts, and under different circumstances. We now have a real chance to rethink which priorities to put at the centre of the political agenda. Global and cross-sectoral governance, as well as policy mechanisms, should be implemented to develop effective measures for removing structural factors that prevent sustainable development.

## THE 2030 AGENDA FOR SUSTAINABLE DEVELOPMENT

An important opportunity to globally address these issues is the 2030 Agenda for Sustainable Development [2]. The 2030 Agenda, adopted by all United Nations Member States (193) in 2015, is made up of 17 Sustainable Development Goals (SDGs) and 169 targets with the aim of strengthening collaboration between countries, to build a better and sustainable society by 2030. The rationale for sustainable development at the basis of the 2030 Agenda is the interconnection between all 17 SDGs and the harmonization between 3 main dimensions – economic, social, and ecological [3].

“Good health and well-being” is the third goal and represents the core of the Agenda to which all other goals are connected. Health depends on all other goals that significantly affect health outcomes, and in turn, the achievement of the other goals depends on the level of health. For example, the economic prosperity of the country is closely linked to the health of the population. When the pandemic broke out, everyone was taken by surprise. Once it became clear that the pandemic was a global emergency and that a prompt public health response was needed, the policy response was slow overall.

Six years after the adoption of the 2030 Agenda, we still have a long way to go to achieving SDGs [4]. A profound rethinking is needed on what is meant by sustainable development and social resilience. Depending on the point of view, the balance can change, and the objectives can be different. Only a global and clear vision can lead member states (MSs) to undertake a truly sustainable and equitable transformative process.

## ITALIAN ALLIANCE FOR SUSTAINABLE DEVELOPMENT

ASviS (Italian Alliance for Sustainable Development) was born in Italy in February 2016 with the aim of raising Italian awareness of the importance of the 2030 Agenda and actively working towards the achievement of the SDGs [5]. ASviS currently brings together 300 of the most important civil society institutions and networks, public and private universities, and research centres nationwide. ASviS is organized into work areas, attributable to the 17 SDGs, that actively contribute to the design of policies for the implementation of the 2030 Agenda. The Alliance's working groups meet regularly to assess both the impact of the measures drawn up by the Government in the light of the SDGs and to develop concrete proposals for sustainable development policies to be systematically submitted to political decision-makers. Currently, about 800 experts are working together to actively support reforms and investments to kick-start the economy and build a more resilient, green, and digital state in Italy. Among their activities is the critical analysis of the NRRP. The analysis offers an examination of the strengths and points of attention of the Plan, as well as a list of “system” proposals to accelerate the transition of our country towards a truly sustainable model. At the same time, ASviS also systematically rules on the “Italian Budget Law”. The Report “The Budget Law and Sustainable Development” is an annual publication of the ASviS which aims to assess the impact of the provisions of the Budget Law on the 17 SDGs and to understand how the measures adopted by the government affect collective well-being. The goal is to verify if Italy is orienting its choices in favour of sustainability, to lead our country on a path of sustainable development.

**Figure:**
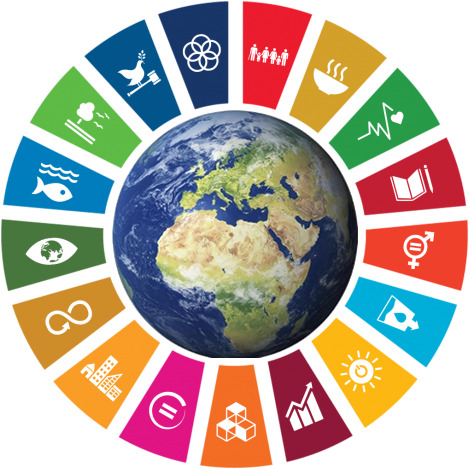
Photo: The 17 UN goals: Sustainable Development goals. Source: Sustainable Development (from https://www.cleanpng.com/png-sustainable-development-goal-1-no-poverty-sustaina-7238309/) and Globe (from https://pixabay.com/it/illustrations/terra-mondo-pianeta-globo-1303628/). No permission was needed for the use of the photographs.

The Sustainable Development Festival, organized annually, is the most important Italian ASviS initiative. Its goal is to raise awareness and mobilize citizens, associations, and institutions on the issues of economic, social, and environmental sustainability, in order to promote a cultural and political change that allows Italy to implement the 2030 Agenda and the 17 SDGs. The Festival was recognized by the UN SDG Action Challenge Awards as an innovative initiative and a unique experience at an international level. In 2020, it was announced among the finalists in the “Mobilize” category [6], while in 2019, the Festival was a finalist in the “Connect” category [7].

To date, it seems that there are no other initiatives like that of the Italian ASviS in other countries. We believe that the ASviS experience could be made more widely known, in order to share this initiative with other European MSs.

## CONCLUSION

In light of the Italian experience, our purpose is to bring together a “European SDGs community” to realize a high-quality, effective, and sustainable European Partnership for promoting SDGs implementation which engages public authorities, civil society, research bodies, and other key actors.

What we recommend is a two-step approach. The first step consists of the implementation of a structure similar to ASviS at a single-country level. The second step is the implementation of international governance composed of the national representatives of each “local ASviS”, who act collectively to achieve the objectives. The SDGs community could contribute to the definition of a national strategy for the implementation of the SDGs thanks to the strengthening of a cooperative approach, the exchange and learning between the MSs, as well as the transferability of good practices. In addition, it could improve the planning and development of the policies to address the SDGs in Europe at large.
